# How Does Domain Replacement Affect Fibril Formation of the Rabbit/Human Prion Proteins

**DOI:** 10.1371/journal.pone.0113238

**Published:** 2014-11-17

**Authors:** Xu Yan, Jun-Jie Huang, Zheng Zhou, Jie Chen, Yi Liang

**Affiliations:** State Key Laboratory of Virology, College of Life Sciences, Wuhan University, Wuhan, China; INRA, France

## Abstract

**Background:**

It is known that *in vivo* human prion protein (PrP) have the tendency to form fibril deposits and are associated with infectious fatal prion diseases, while the rabbit PrP does not readily form fibrils and is unlikely to cause prion diseases. Although we have previously demonstrated that amyloid fibrils formed by the rabbit PrP and the human PrP have different secondary structures and macromolecular crowding has different effects on fibril formation of the rabbit/human PrPs, we do not know which domains of PrPs cause such differences. In this study, we have constructed two PrP chimeras, rabbit chimera and human chimera, and investigated how domain replacement affects fibril formation of the rabbit/human PrPs.

**Methodology/Principal Findings:**

As revealed by thioflavin T binding assays and Sarkosyl-soluble SDS-PAGE, the presence of a strong crowding agent dramatically promotes fibril formation of both chimeras. As evidenced by circular dichroism, Fourier transform infrared spectroscopy, and proteinase K digestion assays, amyloid fibrils formed by human chimera have secondary structures and proteinase K-resistant features similar to those formed by the human PrP. However, amyloid fibrils formed by rabbit chimera have proteinase K-resistant features and secondary structures in crowded physiological environments different from those formed by the rabbit PrP, and secondary structures in dilute solutions similar to the rabbit PrP. The results from transmission electron microscopy show that macromolecular crowding caused human chimera but not rabbit chimera to form short fibrils and non-fibrillar particles.

**Conclusions/Significance:**

We demonstrate for the first time that the domains beyond PrP-H2H3 (β-strand 1, α-helix 1, and β-strand 2) have a remarkable effect on fibrillization of the rabbit PrP but almost no effect on the human PrP. Our findings can help to explain why amyloid fibrils formed by the rabbit PrP and the human PrP have different secondary structures and why macromolecular crowding has different effects on fibrillization of PrPs from different species.

## Introduction

Transmissible spongiform encephalopathies, also known as prion diseases, are infectious fatal neurodegenerative diseases that affect the nervous system in humans and animals [1]. The key procedure of prion diseases is believed to be the conversion of the normal protease-sensitive cellular prion protein (PrP^C^) in such mammals into the aberrant protease-resistant pathogenic prion protein (PrP^Sc^) [1]–[4]. Rabbits are among the few animal species that have resistance to prions from other animal species [5]. However, the three-dimensional structure of the recombinant protein rabbit PrP^C^ is composed of an unstructured flexible N-terminal region and a C-terminal globular domain which comprises two short anti-parallel β-strands and three α-helices similar to the structures of other mammalian PrP^C^ [6]–[8]. There are few difference among the C-terminal domains, and some research believes that the unique primary structure of rabbit PrP^C^ inhibits formation of its abnormal isoform [6], [9].

A cell-free conversion system for reconstitution of the infectious PrP^Sc^ from recombinant PrP *in vitro* has been developed [10]–[12]. The Baskakov lab has provided the first demonstration that recombinant full-length prion protein (PrP) with an intact S-S bond can be folded into amyloid conformation *in vitro* [13]. Using a serial protein misfolding cyclic amplification (PMCA) protocol [14], a prion has been generated with bacterially expressed recombinant PrP in the presence of synthetic anionic phospholipid and RNA [15]. Furthermore, Kim and co-workers have demonstrated that mammalian prions can also be generated from bacterially expressed PrP in the absence of any mammalian cofactors [16]. De novo rabbit prions have been produced by rabbit brain homogenate *in vitro* from unseeded material [17]. All of these works suggest that amyloid fibrils generated *in vitro* are infectious something like PrP^Sc^ and there could be some similarities between them. The Baskakov lab has also provided compelling evidence that noninfectious amyloids with a structure different from that of PrP^Sc^ could lead to transmissible prion diseases and raised a new mechanism responsible for prion diseases [18], which is different from the PrP^Sc^-templated mechanism [19] or spontaneous conversion of PrP^C^ into PrP^Sc^ [20].

Amyloidogenic proteins form fibril deposits in crowded physiological environments [21]–[28]. It is known that *in vivo* human PrP have the tendency to form fibril deposits and are associated with infectious fatal prion diseases [1], [4], while the rabbit PrP does not readily form fibrils and is unlikely to cause prion diseases [9], [27], [28]. Although we have previously demonstrated that amyloid fibrils formed by the rabbit PrP and the human PrP have different secondary structures and macromolecular crowding has different effects on fibril formation of the rabbit/human PrPs [27], [28], the molecular mechanism of this phenomenon is not clear, and we do not know which domains of PrPs cause such differences. The Rezaei lab has found that the α-helical 2 and α-helix 3 (H2H3) domain of the mouse PrP forms amyloid fibrils morphologically similar to those obtained for the full-length PrP and generates insoluble proteinase K (PK)-resistant aggregates [29]–[31]. We want to know the role of the H2H3 domain in fibrillization of the rabbit/human PrPs in crowded physiological environments.

In this study, we constructed two PrP chimeras, in one of which the H2H3 domain of the human PrP was replaced with that of the rabbit PrP (termed rabbit chimera) and in another of which the H2H3 domain of the rabbit PrP is replaced with that of the human PrP (termed human chimera), and used site-directed mutation to analysis how domain replacement affects fibril formation of the rabbit/human PrPs. As revealed by thioflavin T (ThT) binding assays and Sarkosyl-soluble SDS-PAGE, the addition of high concentrations of crowding agents (Ficoll 70 or Ficoll 400) significantly enhanced fibril formation of both PrP chimeras. As evidenced by circular dichroism (CD), Fourier transform infrared (FTIR) spectroscopy, and PK digestion assays, the overall secondary structure and PK resistance of the fibrils formed by human chimera are similar to those formed by the human PrP. However, amyloid fibrils formed by rabbit chimera had PK-resistant features and secondary structures in crowded physiological environments different from those formed by the rabbit PrP, and secondary structures in dilute solutions similar to the rabbit PrP. We also demonstrated that the amino acids beyond PrP-H2H3 had almost no effect on fibrillization of the human PrP but a remarkable effect on the rabbit PrP.

## Materials and Methods

### Ethics statement

All research involving original human work was approved by the Institutional Review Board of the College of Life Sciences, Wuhan University (Wuhan, China), leaded by Dr. Bao-Liang Song, the Dean of the college, in accordance with the guidelines for the protection of human subjects. Written informed consent for the original human work that produced the plasmid samples was obtained. This study was approved by the ethics committee of the College of Life Sciences, Wuhan University, leaded also by Dr. Bao-Liang Song. The vector pET-30a (+) contain mature human prion protein(23–231) (pET-30-hPrP) and the vector pET-30a (+) contain mature rabbit prion protein(23–228) (pET-30-rPrP) were kindly donated by Prof. Geng-Fu Xiao (Wuhan Institute of Virology, Chinese Academy of Sciences). Written informed consent for using and modifying these plasmids was obtained. The plasmids of pET-28a-hH2H3 and pET-28a-rH2H3 were constructed from pET-30-hPrP/pET-30-rPrP and pET-28a (+) by ourselves. All the proteins (human PrP, rabbit PrP, human chimera, rabbit chimera, chimera H, chimera R, human PrP-H2H3, and rabbit PrP-H2H3) involved in this work were produced by *E. coli* BL21 (DE3) expression system. There is not any animal work or *in vivo* experiments performed in this study.

### Materials

The crowding agents, Ficoll 70 and Ficoll 400, were purchased from Sigma-Aldrich (St. Louis, MO). ThT was also obtained from Sigma-Aldrich. Guanidine hydrochloride (GdnHCl) was obtained from Promega (Madison, WI). Proteinase K and Triton X-100 were purchased from Ameresco (Solon, OH). All other chemicals used were made in China and were of analytical grade.

### Plasmid construction and prion protein purification

Standard cloning procedures were used. Using KOD-Plus-Mutagenesis Kit, the H2H3 of the human PrP was replaced by the H2H3 of the rabbit PrP, termed rabbit chimera, which just mutated the sites 174N, 184I, 203V, 205M, 219E, 220R, 225Y, 229G and 230S of human PrP to 174S, 184V, 203I, 205I, 219Q, 220Q, 225A, 229A and 230Stop, using pET-30-hPrP as template. The primers used can be seen in Table S1. The H2H3 of the rabbit PrP was replaced by the H2H3 of the human PrP, termed human chimera, in the same way with primers shown in Table S2, using pET-30-rPrP as template.

The primers used to construct chimera H in which the human PrP-B1H1B2 (β-strand 1, α-helix 1, and β-strand 2) was replaced by the rabbit PrP-B1H1B2 is shown in Table S3, and the primers used to construct chimera R in which the rabbit PrP-B1H1B2 was replaced by the human PrP-B1H1B2 is shown in Table S4.

Human PrP-H2H3 (169–231) was amplified by polymerase chain reaction (PCR) using pET-30-hPrP as template with primers:

SH-H2H3 5′GGGTAATCCATATGTACAGCAACCAGAAC

AH-H2H3 5′ ACAGAATTCTCATCACGATCCTCTCTGGT

The PCR product was cloned between restriction sites Nde I and EcoR I of the vector pET-28a (+), termed pET-28a-hH2H3.

Rabbit PrP-H2H3 (168–228) was amplified by PCR using pET-30-rPrP as template with primers:

SR-H2H3 5′ GGGTGCTCATATGTACAGCAACCAGAACAG

AR-H2H3 5′ ACAGAATTCTTATGCCGCCCTCTGGTAGGC

The PCR product was cloned between restriction sites Nde I and EcoR I of the vector pET-28a (+), termed pET-28a-rH2H3.

Recombinant full-length prion proteins, human chimera, rabbit chimera, chimeras H and R, and PrP-H2H3 were expressed in *E. coli* BL21 (DE3) (Novagen), and purified by HPLC on a C4 reversed-phase column (Shimadzu, Kyoto, Japan) as described by Bocharova and co-workers [32].

### Thioflavin T binding assays

The methods for fibrillization of full-length prion proteins, human chimera, and rabbit chimera were the same as the methods described by the Liang lab [28], and 10 µM PrP was incubated at 37°C in PBS buffer (pH 7.0) containing 2 M GdnHCl in the absence and presence of crowding agents with continuous shaking at 220 rpm, and samples (50 µl) were diluted into PBS buffer containing 12.5 µM ThT, giving a final volume of 2.5 ml.

The method for fibrillization of PrP-H2H3 was similar to the method described by the Rezaei lab [30]. Because urea is not stable at pH 5.0, we modified the method as follow: 10 µM PrP-H2H3 was incubated at 37°C in 20 mM NaAc buffer (pH 5.0) containing 200 mM NaCl and 1 M GdnHCl in the absence and presence of crowding agents with continuous shaking at 220 rpm, and samples (50 µl) were diluted into NaAc buffer buffer containing 12.5 µM ThT, giving a final volume of 2.5 ml.

As described in detail previously [28], [33]–[35], the fluorescence of ThT was excited at 450 nm (slit-width, 5 nm) and the emission was measured at 480 nm (slit-width, 5 nm) for PrPs on an LS-55 luminescence spectrometer (PerkinElmer Life Sciences, Shelton, CT). The fluorescence intensity at 480 nm was averaged over 60 s to increase the signal-to-noise ratio of the measurements. Control experiments were performed to ensure that the crowding agents had no influence on the ThT binding assays for PrPs. Kinetic parameters were determined by fitting ThT fluorescence intensity *versus* time to a sigmoidal equation, as described in detail previously [25], [27], [33], [36], [37]:
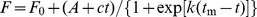
(1)where *F* is the fluorescence intensity, *k* the rate constant for the growth of fibrils, and *t_m_* the time to 50% of maximal fluorescence. *F*_0_ describes the initial baseline during the lag time. *A+ct* describes the final baseline after the growth phase has ended. The lag time is determined to be *t_m_*−2/*k*.

### Sarkosyl-soluble SDS-PAGE

The protocol for Sarkosyl-soluble SDS-PAGE was described in detail previously [28], [33]. Briefly, amyloid formation of 10 µM PrP was carried out as state above, during the incubation time, 20 µl samples were taken out and added with 2.5 µl of 100 mM Tris-HCl (pH 7.0) and 2.5 µl of 20% Sarkosyl. The mixture were left at room temperature for 30 min, then mixed with 2× loading buffer (without SDS and no heating) and separated by 15% SDS-PAGE. Gels were stained by Coomassie Blue.

### Transmission electron microscopy

**Figure 1:**
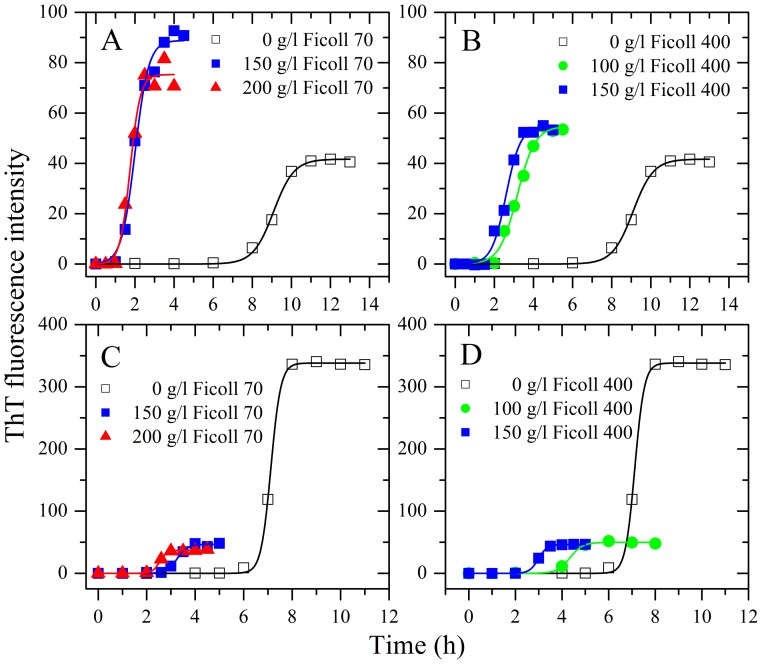
Effects of macromolecular crowding on amyloid formation of human chimera and rabbit chimera of prion proteins. Human chimera (A and B) and rabbit chimera (C and D) in the absence and presence of Ficoll 70 (A and C) or Ficoll 400 (B and D), monitored by ThT fluorescence. All experiments were repeated at least three times. The final concentrations of all prion proteins were 10 µM. The crowding agent concentrations were 0 (open square), 100 g/l (solid circle), 150 g/l (solid square), and 200 g/l (solid triangle), respectively. A sigmoidal equation was fitted to the data and the solid lines represented the best fit. The corresponding kinetic parameters from A–D are summarized in Table 1.

The formation of fibrils by PrPs was confirmed by electron microscopy of negatively stained samples. The preparation for negatively stained samples was described in detail previously [28], [33]–[35]. Briefly, the incubation time was chosen within a time range of the plateau of each kinetic curve of ThT fluorescence shown in Fig. 1. Sample aliquots of 10 µl were placed on copper grids and left at room temperature for 1–2 min, rinsed twice with H_2_O, and then stained with 2% (w/v) uranyl acetate for another 1–2 min. The stained samples were examined using an FEI Tecnai G2 20 transmission electron microscope (Hillsboro, OR) operating at 200 kV or an H-8100 transmission electron microscope (Hitachi, Tokyo, Japan) operating at 100 kV.

### CD measurements

Circular dichroism spectra were obtained by using a Jasco J-810 spectropolarimeter (Jasco Corp., Tokyo, Japan) with a thermostated cell holder, as described in detail previously [34], [35]. Briefly, quartz cell with a 1 mm light-path was used for measurements in the far-UV region. Spectra were recorded from 195 to 250 nm for far-UV CD. PrP fibril samples were subjected to extensive dialysis against NaAc buffer (pH 5.0) to remove guanidine hydrochloride. The final concentration of PrP was kept at 10 µM. The averaged spectra of several scans were corrected relative to the buffer blank or the buffer containing crowding agents. Measurements were made at 25°C. All CD experiments were repeated three times. The experiments were pretty reproducible.

### Fourier transform infrared spectroscopy

Attenuated total reflection FTIR spectra of PrP fibril samples were recorded using a Nicolet 5700 FTIR spectrophotometer (Thermo Electron, Madison, WI), as described in detail previously [27]. Briefly, 3 ml PrP fibril samples were harvested (∼150000 g) within a time range of the plateau of each kinetic curve of ThT fluorescence, and then washed by H_2_O and dried by vacuum drying. The dried samples were prepared in D_2_O and FTIR spectra were recorded in the range from 400 to 4000 cm^−1^ at 4 cm^−1^ resolution. The sample was scanned 32 times in each FTIR measurement, and the spectrum acquired is the average of all these scans. Spectra were corrected for the D_2_O and water vapors. Measurements were made at 25°C.

### PK digestion assays

PrP fibril samples were prepared in 100 mM Tris-HCl buffer (pH 7.5) and incubated with PK at a PK: PrP molar ratio of 1∶100 to 1∶50 for 1 h at 37°C. Digestion was stopped by the addition of 2 mM phenylmethylsulfonyl fluoride (PMSF), and samples were analyzed in 15% SDS-PAGE and detected by silver staining.

## Results

### Amyloid fibrils formed by human chimera had secondary structures and PK-resistant features similar to those formed by the human PrP

Our previous studies [27], [28] have shown that macromolecular crowding significantly accelerated fibril formation of the human PrP. In this study, the effects of nominally inert polymeric additives, Ficoll 70 and Ficoll 400, on kinetics of amyloid fibril formation of the recombinant human PrP chimera were examined by ThT binding assays (Fig. 1, A and B), as a function of crowder concentration.

As shown in Fig. 1A, the presence of Ficoll 70 at concentrations of 150 g/l and 200 g/l in PBS buffer (pH 7.0) containing 2 M GdnHCl significantly accelerated amyloid fibril formation of human chimera on the investigated time scale, accompanied by a remarkable increase in the maximum ThT intensity, which is very similar to that of the human PrP under the same conditions [28]. As shown in Fig. 1B, the presence of Ficoll 400 at concentrations of 100 g/l and 150 g/l also strongly enhanced fibril formation of human chimera on the investigated time scale, accompanied by an increase in the maximum ThT intensity. To elucidate the detailed effects of macromolecular crowding on amyloid fibril formation of human chimera, a sigmoidal equation was used to fit the kinetic data, yielding three kinetic parameters which are summarized in Table 1. As shown in Table 1, the addition of 150 g/l Ficoll 70 dramatically accelerated both nucleation and elongation steps of human chimera fibrillization, resulting in a lag time of 1.31 h in the presence of Ficoll 70, which is 6.1-fold decreased compared with that in the absence of a crowding agent (8.05 h), and a rate constant for the growth of fibrils of 2.89 h^−1^, which is 1.5-fold larger than that in the absence of a crowding agent (1.92 h^−1^). Similarly, the addition of 150 g/l Ficoll 400 also markedly accelerated both steps of human chimera fibrillization (Table 1).

**Table 1:** Kinetic parameters of amyloid formation of human chimera or rabbit chimera in the absence and presence of Ficoll 70 or Ficoll 400 by ThT binding assays at 37°C.

PrP chimera	Crowding agent	*A*	Lag time (h)	*k* (h^−1^)
Human chimera	0	41.7±0.7	8.05±0.15	1.92±0.18
	150 g/l Ficoll 70	88.8±2.7	1.31±0.17	2.89±0.45
	200 g/l Ficoll 70	75.3±2.5	1.23±0.16	3.83±0.72
	100 g/l Ficoll 400	54.7±1.5	2.26±0.16	2.19±0.23
	150 g/l Ficoll 400	54.5±1.4	1.86±0.14	2.74±0.32
Rabbit chimera	0	338±2	6.72±0.09	5.01±0.88
	150 g/l Ficoll 70	46.7±1.3	2.82±0.10	4.66±0.63
	200 g/l Ficoll 70	37.1±0.4	2.24±0.06	6.88±1.09
	100 g/l Ficoll 400	49.8±0.8	3.79±0.12	3.70±0.47
	150 g/l Ficoll 400	46.5±0.1	2.61±0.01	5.31±0.15

Best-fit values of these kinetic parameters were derived from non-linear least squares modeling of a sigmoidal equation as described in the “Materials and Methods” to the data plotted in Fig. 1. Errors shown are ± S.E.

**Figure 2:**
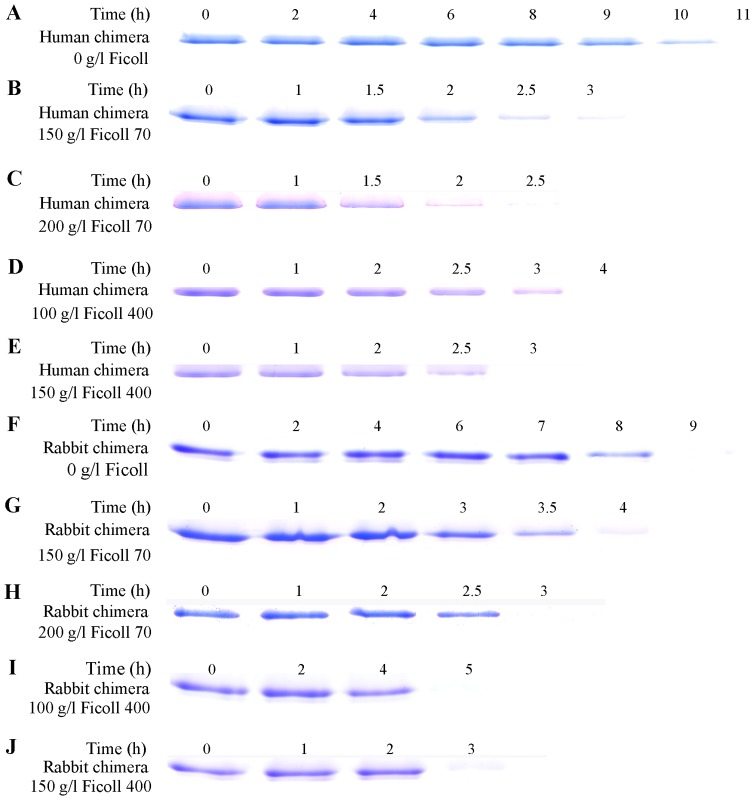
Time-dependent SDS-PAGE analysis of Sarkosyl-soluble human chimera (A–E) and rabbit chimera (F–J) of prion proteins incubated in 0 g/l Ficoll (A and F), 150 g/l (B and G) and 200 g/l (C and H) Ficoll 70, and 100 g/l (D and I) and 150 g/l (E and J) Ficoll 400. We took and dialyzed the samples against 10 mM Tris-HCl (pH 7.0), and incubated them with 10 mM Tris-HCl buffer containing 2% Sarkosyl for 30 min. Then we centrifugated the samples at 17,000 g for 30 min and mixed the supernatants with 2× loading buffer and separated them by 15% SDS-PAGE. The human/rabbit PrPs were denatured in PBS buffer (pH 7.0) containing 2 M GdnHCl.

After centrifugation assays, Sarkosyl-soluble SDS-PAGE experiments were carried out to semi-quantify the decrease of PrP monomers in the presence of crowders. As shown in Fig. 2, A–E, when human chimera was incubated in the absence of a crowding agent for 10 h, a clear band corresponding to Sarkosyl-soluble human chimera monomers was observed, while when human chimera was incubated with 150 g/l Ficoll 70/Ficoll 400 for 2.5–3 h, a much shorter time than 10 h, the human chimera monomer band was observed. The above results indicate that crowding agents dramatically promoted fibril formation of human chimera, similar to that of the human PrP [28].

**Figure 3:**
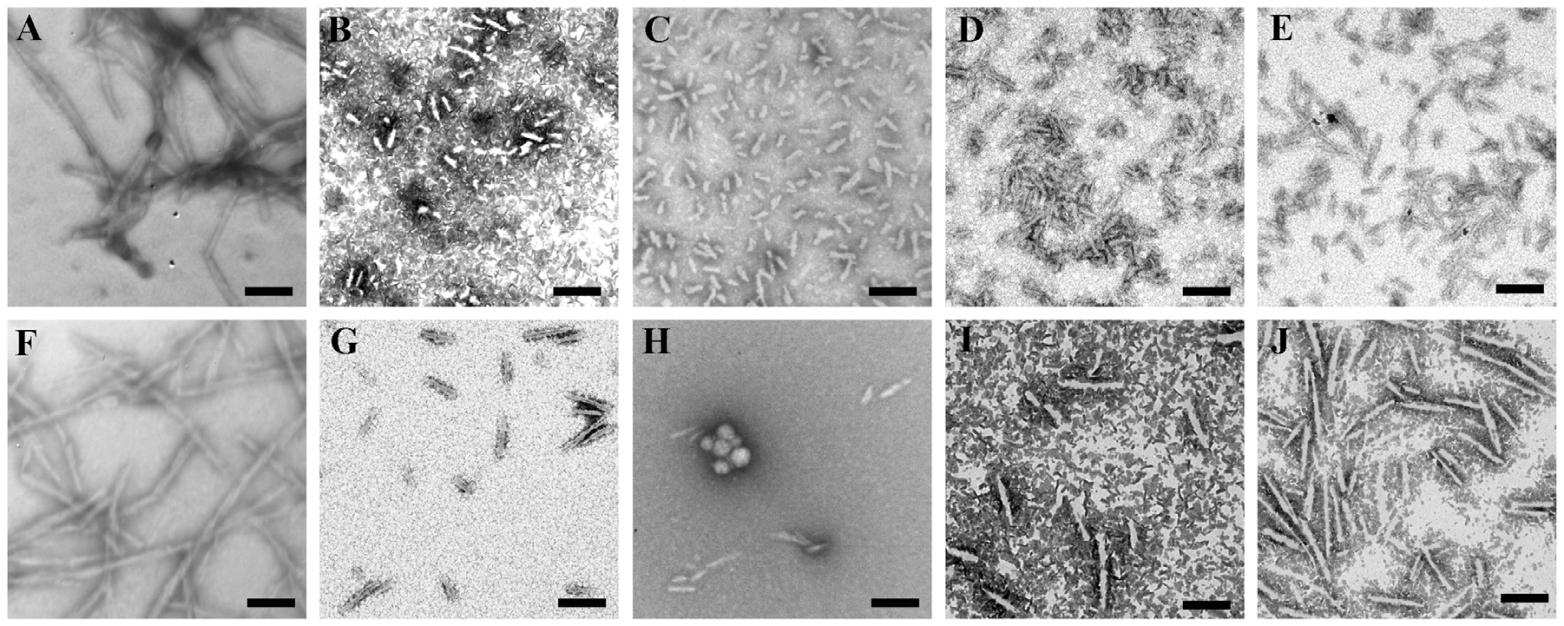
Transmission electron micrographs of human/rabbit chimera PrP samples at physiological pH after incubation under different conditions. Human (A–E) and rabbit (F–J) chimera PrP samples were incubated for 10 h (A) or 8 h (F) or 4 h (D, G, and I) or 3 h (B, E, H, and J) or 2 h (C) in the absence of a crowding agent (A and F) and in the presence of 150 g/l Ficoll 70 (B and G) or 200 g/l Ficoll 70 (C and H) or 100 g/l Ficoll 400 (D and I) or 150 g/l Ficoll 400 (E and J), respectively. We used a 2% (w/v) uranyl acetate solution to stain the fibrils negatively. The scale bars represent 200 nm.

TEM was then employed to characterize the morphology of human chimera aggregates formed in the absence and in the presence of crowding agents. Fig. 3, A–E, shows TEM images of human chimera samples incubated in the solution of a crowding agent (Ficoll 70 or Ficoll 400), compared with those in dilute solutions. In absence of a crowding agent, human chimera formed fibrils with a long, twisted, and branched structure after incubation for 10 h (Fig. 3A), which is similar to those produced from the human PrP or the bovine PrP in the absence of a crowding agent [25], [27]. In the presence of 150 g/l, 200 g/l Ficoll 70 or 100 g/l, 150 g/l Ficoll 400, however, abundant short fibrillar fragments and spherical or ellipsoidal particles were observed when human chimera samples were incubated for 2–4 h (Fig. 3, B–E), which is similar to those produced from the human PrP or the bovine PrP in the presence of 150 g/l Ficoll 70 [25], [27]. The above results indicated that macromolecular crowding also caused human chimera to form short fibrils and non-fibrillar particles, which is very similar to that of the human PrP or the bovine PrP [25], [27].

**Figure 4:**
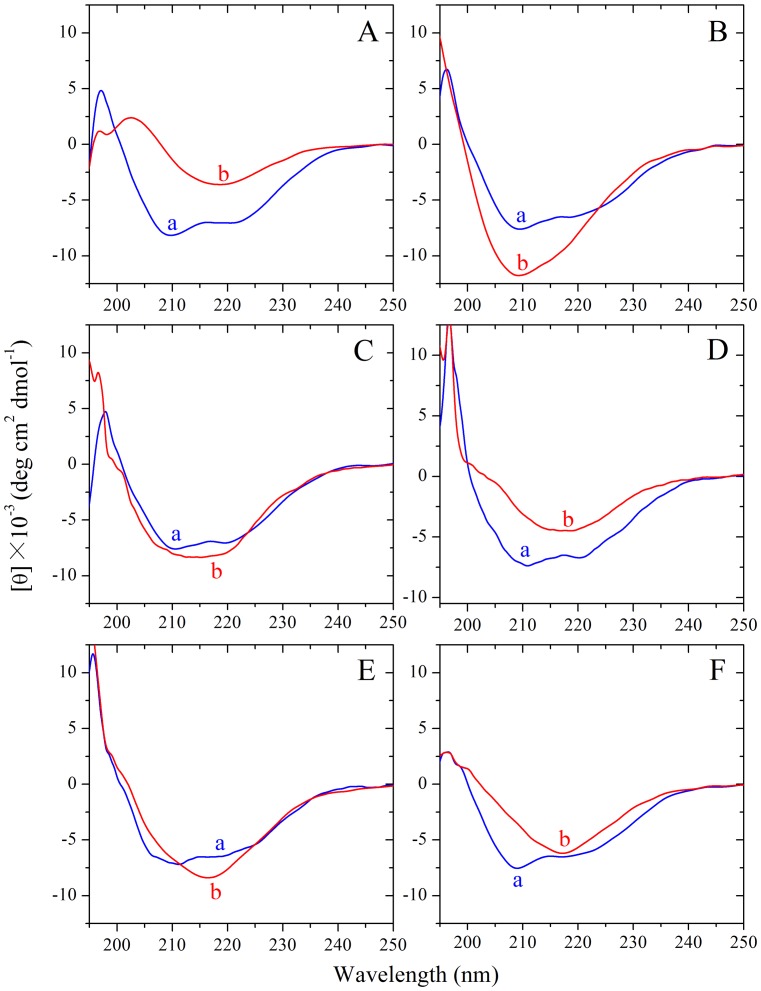
Secondary structural changes of human (A, C, and E) and rabbit (B, D, and F) chimera PrP isoforms monitored by far-UV CD. Curve a: native human/rabbit chimera prion proteins, and Curve b: amyloid fibrils produced from human chimera and rabbit chimera, in the absence of a crowding agent (A and B) and in the presence of 150 g/l Ficoll 70 (C and D) or 100 g/l Ficoll 400 (E and F), respectively. The incubation time was chosen within a time range of the plateau of each kinetic curve of ThT fluorescence shown in Fig. 1.

CD spectroscopy was used to confirm the formation of amyloid fibrils by human chimera. Fig. 4, A, C, and E, shows the CD spectra of native human chimera and human chimera fibrils formed in the absence and presence of 150 g/l Ficoll 70 or 100 g/l Ficoll 400. Under all conditions, human chimera formed amyloid fibrils with β-sheet-rich conformation (a single minimum around 218 nm was observed) from the native state which has predominant α-helix conformation with double minima at 208 and 222 nm.

**Figure 5:**
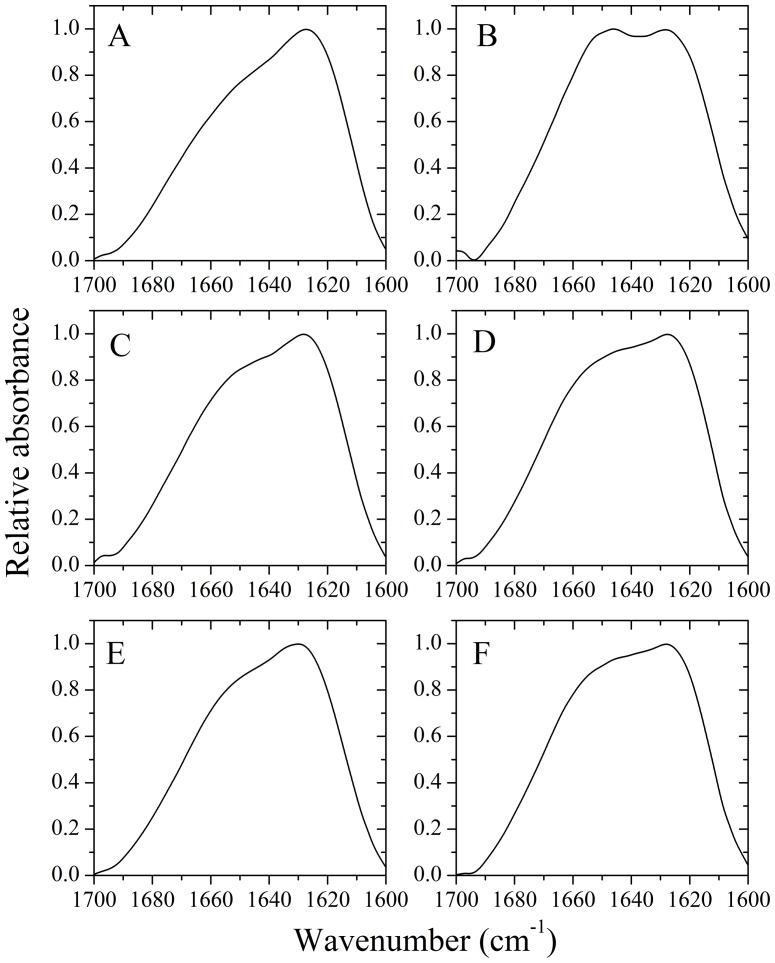
Secondary structural changes of human (A, C, and E) and rabbit (B, D, and F) chimera PrP isoforms monitored by FTIR. The FTIR spectra of amyloid fibrils produced from human chimera and rabbit chimera, in the absence of a crowding agent (A and B) and in the presence of 150 g/l Ficoll 70 (C and D) or 100 g/l Ficoll 400 (E and F), respectively. The incubation time was chosen within a time range of the plateau of each kinetic curve of ThT fluorescence shown in Fig. 1.

CD is not the best approach to investigate the secondary structure of hyper-large protein assemblies as at this scale anisotropic light scattering could take place. The shape and position of amide I′ (1600–1700 cm^−1^) of FTIR bands provide detailed information on the secondary structure of proteins, and the amide I′ band at 1630 cm^−1^ is characteristic for β-sheet formed by amyloid fibrils [27]. Considering this, we performed FTIR experiments to investigate the secondary structure of amyloid fibrils formed by human chimera. Fig. 5, A, C, and E, shows the FTIR spectra in the amide I′ region of human chimera fibrils formed in the absence and presence of 150 g/l Ficoll 70 or 100 g/l Ficoll 400. Under all conditions, human chimera formed amyloid fibrils with β-sheet-rich conformation (a single amide I′ band around 1630 cm^−1^ was observed).

**Figure 6:**
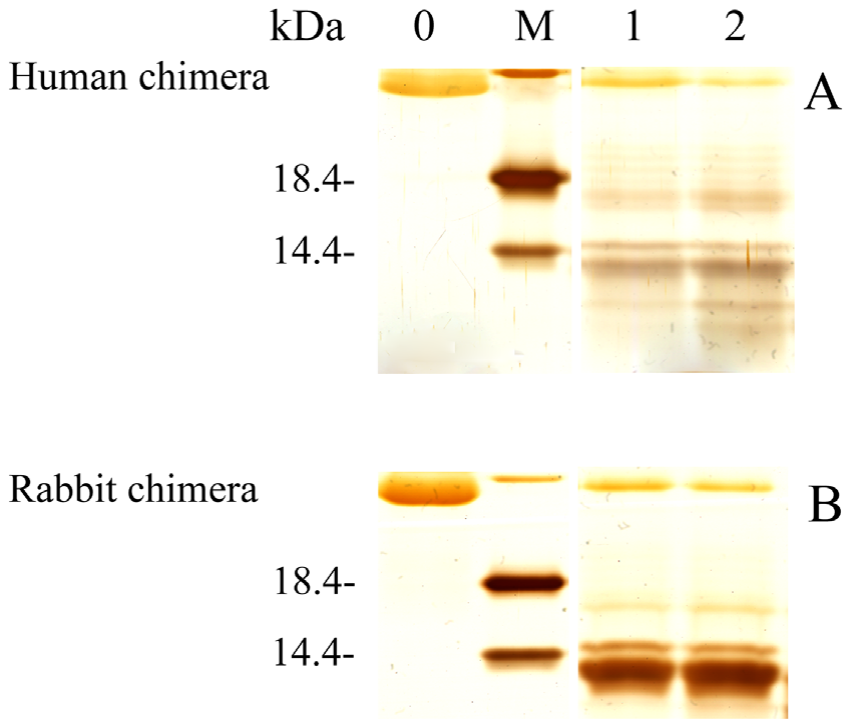
Concentration-dependent proteinase K-digestion assays of human (A) and rabbit (B) chimera PrP fibrils. Samples were treated with PK for 1 h at 37°C at PK: PrP molar ratios as follows: 1∶100 (lane 1) and 1∶50 (lane 2). PK concentration: 0.4 µg/ml (lane 1) and 0.8 µg/ml (lane 2). The controls with zero protease in the absence of a crowding agent were loaded in lane 0. Protein molecular weight markers were loaded on lane M: restriction endonuclease Bsp98 I (25.0 kDa), β-lactoglobulin (18.4 kDa), and lysozyme (14.4 kDa). Amyloid fibrils were produced from human chimera and rabbit chimera in the absence of a crowding agent. Protein fragments were separated by SDS-PAGE and detected by silver staining.

PK resistance activity has been widely used to distinguish PrP^C^ from PrP^Sc^ since the pioneering studies of Prusiner and co-workers [38]. As shown in Fig. 6A, amyloid fibrils produced from human chimera generated PK-resistant fragments of 15–16-kDa after PK digestion for 1 h, which are similar to those of the human PrP [25], [27]. Human chimera fibrils also generated three short fragments (12-, 10-, and 8-kDa bands), which are similar to those of the human PrP reported previously [25], [27]. Taken together, our CD, FTIR and PK digestion data demonstrate that amyloid fibrils formed by human chimera have secondary structures and PK-resistant features similar to those formed by the human PrP.

### Amyloid fibrils formed by rabbit chimera had PK-resistant features and secondary structures in crowded physiological environments different from those formed by the rabbit PrP

We have demonstrated previously that macromolecular crowding remarkably inhibited fibril formation of the rabbit PrP [27], [28]. In this study, the effects of two macromolecular crowding agents, Ficoll 70 and Ficoll 400, on kinetics of amyloid fibril formation of the recombinant rabbit PrP chimera were examined by ThT binding assays (Fig. 1, C and D), as a function of crowder concentration.

To our surprise, the presence of Ficoll 70 at concentrations of 150 g/l and 200 g/l significantly accelerated the nucleation step of fibril formation of rabbit chimera (a significant decrease in the lag time) (Fig. 1C), which is different from that of the rabbit PrP under the same conditions [27], [28], but accompanied by a decrease of one order of magnitude in the maximum ThT intensity (Fig. 1C), which is similar to that of the rabbit PrP [27], [28]. As shown in Fig. 1D, the presence of Ficoll 400 at concentrations of 100 g/l and 150 g/l also strongly enhanced the nucleation step of fibril formation of rabbit chimera but accompanied by a decrease of one order of magnitude in the maximum ThT intensity. Clearly, the impact of crowding agents on fibril formation of rabbit chimera is more dramatic than that of human chimera. To elucidate the detailed effects of macromolecular crowding on amyloid fibril formation of rabbit chimera, a sigmoidal equation was used to fit the kinetic data, yielding three kinetic parameters which are also summarized in Table 1. As shown in Table 1, the addition of 150 g/l Ficoll 70 remarkably accelerated the nucleation step of rabbit chimera fibrillization, resulting in a lag time of 2.82 h in the presence of Ficoll 70, which is 2.4-fold decreased compared with that in the absence of a crowding agent (6.72 h), and the addition of 150 g/l Ficoll 400 also markedly accelerated the nucleation step of rabbit chimera fibrillization. However, the addition of 150 g/l Ficoll 70 or 150 g/l Ficoll 400 significantly decreased the amount of rabbit chimera fibrils represented by *A* (Table 1).

In order to confirm the enhancing effect of macromolecular crowding on the nucleation step of fibril formation of rabbit chimera, we carried out Sarkosyl-soluble SDS-PAGE experiments. As shown in Fig. 2, F–J, when rabbit chimera was incubated in the absence of a crowding agent for 8 h, a clear band corresponding to Sarkosyl-soluble rabbit chimera monomers was observed, while when rabbit chimera was incubated with 150 g/l Ficoll 70/Ficoll 400 the band disappeared within 4–5 h. The above results indicate that crowding agents remarkably promoted the nucleation step of fibril formation of rabbit chimera, different from that of the rabbit PrP [27], [28].

The morphology of rabbit chimera aggregates formed in the absence and in the presence of crowding agents was characterized by using TEM. Fig. 3, F–J, shows TEM images of rabbit chimera samples incubated in the solution of a crowding agent (Ficoll 70 or Ficoll 400), compared with those in dilute solutions. In dilute solutions, rabbit chimera formed long and branched fibrils after incubation for 8 h (Fig. 3F). However, rabbit chimera formed some short amyloid fibrils and spherical particles when incubated with 150–200 g/l Ficoll 70 or 100–150 g/l Ficoll 400 for 3–4 h (Fig. 3, G–J). The amount of fibrils formed by rabbit chimera incubated with crowding agents (Fig. 3, G–J) was remarkably less than that in dilute solutions (Fig. 3F) on the same time scale, consistent with the conclusion from ThT binding assays that macromolecular crowding significantly decreased the amount of rabbit chimera fibrils.

CD spectroscopy was used to confirm the significant differences in secondary structures between rabbit chimera fibrils formed in crowded physiological environments and those formed in dilute solutions. Fig. 4, B, D, and F, shows the CD spectra of native rabbit chimera and rabbit chimera fibrils formed in the absence and presence of 150 g/l Ficoll 70 or 100 g/l Ficoll 400. As shown in Fig. 4, B, D, and F, in the absence and presence of a crowding agent and before incubation, the CD spectra measured for rabbit chimera sample had double minima at 208 and 222 nm, indicative of predominant α-helical structure. After incubation for 4 h, a single minimum around 218 nm was observed for rabbit chimera fibril samples in crowded physiological environments (Fig. 4, D and F), which is typical of predominant β-sheet structure and a characteristic for amyloid formation. After incubation for 8 h, however, a single minimum around 210 nm (but not 218 nm) was observed for rabbit chimera fibril samples in dilute solutions (Fig. 4B), which is similar to that of the rabbit PrP in dilute solutions [27]. Then we performed FTIR experiments to investigate the secondary structure of amyloid fibrils formed by rabbit chimera. Fig. 5, B, D, and F, shows the FTIR spectra in the amide I′ region of rabbit chimera fibrils formed in the absence and presence of 150 g/l Ficoll 70 or 100 g/l Ficoll 400. After incubation for 4 h, a single amide I′ band around 1630 cm^−1^ was observed for rabbit chimera fibril samples in crowded physiological environments (Fig. 5, D and F), which is typical of predominant β-sheet structure and a characteristic for amyloid formation. After incubation for 8 h, however, two amide I′ bands around 1650 cm^−1^ and 1630 cm^−1^ were observed for rabbit chimera fibril samples in dilute solutions (Fig. 5B), which is similar to that of the rabbit PrP in dilute solutions [27]. The above data demonstrate that some PrP chimeras could form amyloid fibrils with different structural features in absence and presence of crowding agents, revealing the importance of macromolecular crowding on protein misfolding.

PK digestion assays was used to confirm the significant differences in PK resistance activities between rabbit chimera fibrils and rabbit PrP fibrils. As shown in Fig. 6B, amyloid fibrils produced from rabbit chimera did generate PK-resistant fragments of 15–16-kDa after PK digestion for 1 h, which are different from those of the rabbit PrP but similar to those of the human PrP [25], [27]. Taken together, our CD, FTIR and PK digestion data demonstrate that amyloid fibrils formed by rabbit chimera had PK-resistant features and secondary structures in crowded physiological environments different from those formed by the rabbit PrP.

### Amyloid formation of human PrP-H2H3 and rabbit PrP-H2H3

**Figure 7:**
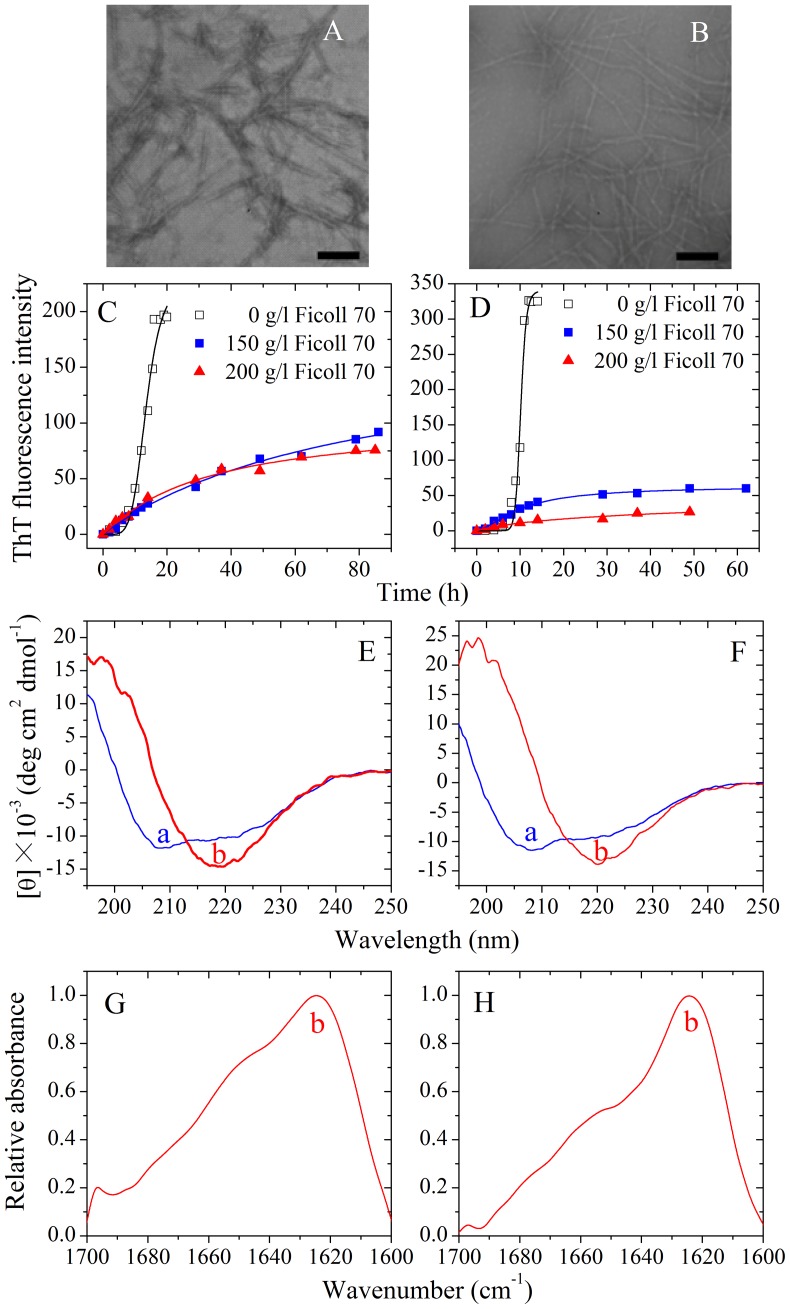
Amyloid formation of human PrP-H2H3 and rabbit PrP-H2H3. Transmission electron micrographs of human PrP-H2H3 (A) and rabbit PrP-H2H3 (B) were made after incubation under different conditions. PrP-H2H3 samples were incubated in the absence of a crowding agent for 14 h (A) and 10 h (B), respectively. Fibril formation of human PrP-H2H3 (C) and rabbit PrP-H2H3 (D) in the absence and in the presence of Ficoll 70, monitored by ThT fluorescence. All experiments were repeated at least three times. The final concentrations of all prion proteins were 10 µM. The crowding agent concentrations were 0 (open square), 150 g/l (solid square), and 200 g/l (solid triangle), respectively. The empirical Hill equation was fitted to the data and the solid lines represented the best fit. Secondary structural changes of human PrP-H2H3 (E and G) and rabbit PrP-H2H3 (F and H) isoforms monitored by far-UV CD and FTIR, respectively. Curve a: native human/rabbit PrP-H2H3. Curve b: amyloid fibrils produced from human PrP-H2H3 and rabbit PrP-H2H3 incubated for 20 h in the absence of a crowding agent.

In order to know the role of the H2H3 domain in amyloid fibril formation of the rabbit/human PrPs, we investigated amyloid formation of human PrP-H2H3 and rabbit PrP-H2H3, by using ThT binding assays, CD, FTIR, and TEM methods. Fig. 7, A and B, shows TEM images of human PrP-H2H3 and rabbit PrP-H2H3 samples. In absence of a crowding agent, both human PrP-H2H3 and rabbit PrP-H2H3 formed fibrils with a long, twisted, and branched structure after incubation for 14 and 10 h, respectively. Fig. 7, C and D, shows the effects of macromolecular crowding on fibril formation of human/rabbit PrP-H2H3 examined by ThT binding assays. To our surprise, the kinetics of fibril formation of both human PrP-H2H3 and rabbit PrP-H2H3 are similar with a similar lag time of 8 h, and the effects of macromolecular crowding on fibril formation of human/rabbit PrP-H2H3 are also similar (Fig. 7, C and D). As shown in Fig. 7, C and D, the presence of Ficoll 70 at concentrations of 150 g/l and 200 g/l significantly accelerated the nucleation step of fibril formation of both human PrP-H2H3 and rabbit PrP-H2H3 (a significant decrease in the lag time) but accompanied by a remarkable decline of the maximum ThT intensity. Fig. 7, E and F, shows the CD spectra of native human/rabbit PrP-H2H3 and human/rabbit PrP-H2H3 fibrils formed in the absence of a crowding agent. Under such conditions, both human PrP-H2H3 and rabbit PrP-H2H3 formed amyloid fibrils with β-sheet-rich conformation (a single minimum around 218 nm was observed) from the native state which has predominant α-helix conformation with double minima at 208 and 222 nm. Fig. 7, G and H, shows the FTIR spectra in the amide I′ region of human/rabbit PrP-H2H3 fibrils formed in the absence of a crowding agent. Under such conditions, both human PrP-H2H3 and rabbit PrP-H2H3 formed amyloid fibrils with β-sheet-rich conformation (a single amide I′ band around 1630 cm^−1^ was observed). Clearly, amyloid formation of human PrP-H2H3 is similar to that of rabbit PrP-H2H3, and the amino acids beyond PrP-H2H3 need to be explored in order to explain why amyloid fibrils formed by the rabbit PrP and the human PrP have different secondary structures and why macromolecular crowding has different effects on fibril formation of the rabbit/human PrPs.

### The amino acids beyond PrP-H2H3 had a remarkable effect on fibrillization of the rabbit PrP

**Figure 8:**
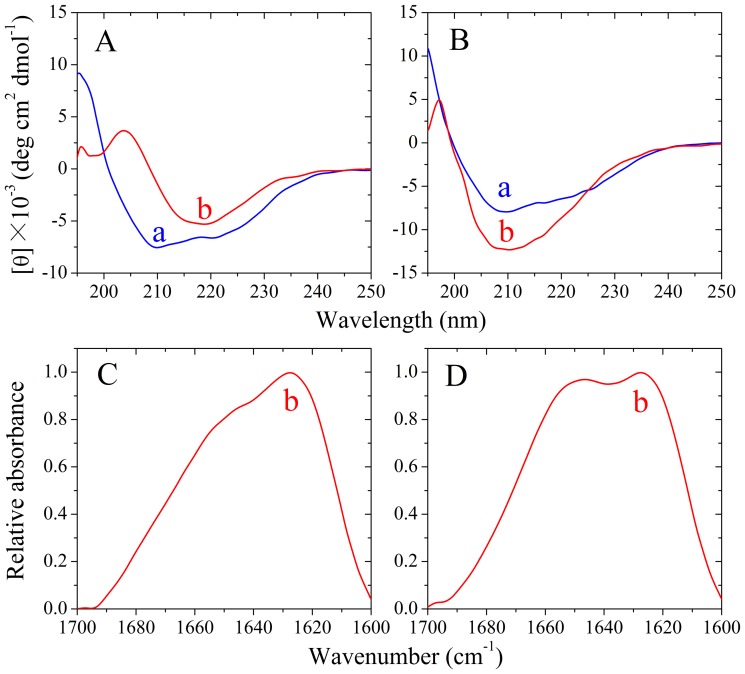
Secondary structural changes of chimera H (A and C) and chimera R (B and D) isoforms monitored by far-UV CD and FTIR, respectively. Curve a: native chimera prion proteins. Curve b: amyloid fibrils produced from chimera prion proteins in the absence of a crowding agent. The incubation time was chosen within a time range of the plateau of each kinetic curve of ThT fluorescence shown in Fig. S1.

**Figure 9:**
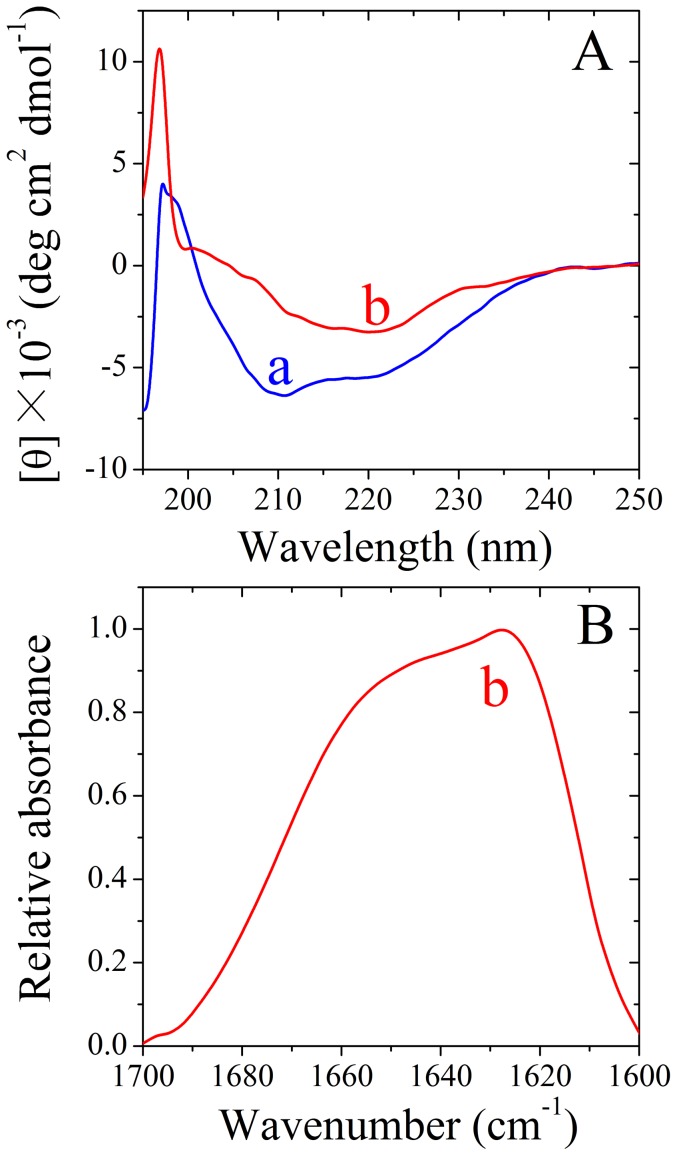
Secondary structural changes of chimera R isoforms in the presence of Ficoll 70 monitored by far-UV CD (A) and FTIR (B). Curve a: native chimera R. Curve b: amyloid fibrils produced from chimera R in the presence of 150 g/l Ficoll 70. The incubation time was chosen within a time range of the plateau of each kinetic curve of ThT fluorescence shown in Fig. S1.

Then we want to know whether the amino acids beyond PrP-H2H3 can influence fibrillization of the rabbit/human PrPs. We thus constructed chimera H, in which the human PrP-B1H1B2 (β-strand 1, α-helix 1, and β-strand 2) domain was replaced by the rabbit PrP-B1H1B2 domain, and chimera R, in which the rabbit PrP-B1H1B2 domain was replaced by the human PrP-B1H1B2 domain, and studied amyloid formation of chimeras H and R, compared with that of the human PrP and the rabbit PrP. As shown in Fig. S1A, the presence of 200 g/l Ficoll 70 significantly accelerated both nucleation and elongation steps of fibril formation of chimera H, which is not only similar to that of the human PrP [28] but also similar to that of human chimera (Fig. 1A). Fig. 8A shows the CD spectra of native chimera H and chimera H fibrils formed in dilute solutions, and Fig. 8C shows the FTIR spectra in the amide I′ region of chimera H fibrils formed in dilute solutions. Similar to human chimera and the human PrP, chimera H also formed amyloid fibrils with β-sheet-rich conformation from the native state which has predominant α-helix conformation. As shown in Fig. S1B, the presence of 200 g/l Ficoll 70 remarkably enhanced the nucleation step of fibril formation of chimera R but accompanied by a very strong decline the maximum ThT intensity, which is similar to that of rabbit chimera (Fig. 1C). CD spectroscopy and FTIR spectroscopy were also employed to confirm the significant differences in secondary structures between chimera R fibrils formed in crowded physiological environments and those formed in dilute solutions. Figs. 8B and 9A show the CD spectra of native chimera R and chimera R fibrils formed in the absence and presence of 150 g/l Ficoll 70, respectively. Figs. 8D and 9B show the FTIR spectra in the amide I′ region of chimera R fibrils formed in the absence and presence of 150 g/l Ficoll 70, respectively. As shown in Figs. 8B and 9A, in the absence and presence of a crowding agent and before incubation, the CD spectra measured for chimera R sample had double minima at 208 and 222 nm, indicative of predominant α-helical structure. After incubation for 3 h, a single minimum around 218 nm or a single amide I′ band around 1630 cm^−1^ was observed for chimera R fibril samples in crowded physiological environments (Fig. 9, A and B), which is typical of predominant β-sheet structure and different from that of the rabbit PrP in crowded physiological environments [27]. After incubation for 5 h, however, a single minimum around 210 nm or two amide I′ bands around 1650 cm^−1^ and 1630 cm^−1^ were observed for chimera R fibril samples in dilute solutions (Fig. 8, B and D), which is similar to not only that of rabbit chimera in dilute solutions (Figs. 4B and 5B) but also that of the rabbit PrP in dilute solutions [27]. The above data once again demonstrate that some PrP chimeras could form amyloid fibrils with different structural features in absence and presence of crowding agents.

## Discussion

Prion protein, a unique infectious amyloid disease-associated protein, causes many lethal human and animal prion diseases [1]. Rabbits are only sensitive to artificial rabbit prion [17] and resistant to prions from other animal species [5]. It has been demonstrated that the unique rabbit PrP sequence, β2-α2 helix-cap, and the residues surrounding the glycosylphosphatidylinositol anchor attachment site could contribute to its resistance to prion diseases [9], [39]–[41]. Furthermore, the presence of either serine (rabbit) or asparagine (hamster) residues in positions 170 and 174 of PrP not only affect the secondary structure of the β2-α2 loop but also the propensity with which the PrP misfolds into β-state-rich octamers [42]. A recent molecular dynamics study has indicated that the sites I214 and S173 of the rabbit PrP can influence the stability of rabbit PrP native state [43]. We have demonstrated previously that macromolecular crowding remarkably inhibits fibril formation of the rabbit PrP but significantly accelerates fibril formation of the human/bovine PrPs [27], [28]. In this study, we want to know which domains of PrPs cause such differences. To align the rabbit PrP23-228 with the human PrP23-231, we found that there are 88% identities between their sequences. Hydrogen/deuterium exchange and solid-state NMR results have demonstrated that PrP fibrils contain in-register parallel β-sheets and that the structurally ordered fibril core includes the C-terminal segment, approximately residues 175–225, which includes the H2H3 of monomeric PrP [44], [45]. In this study the effects of replacement certain domains in rabbit and human PrPs on fibril formation under natural crowding conditions were investigated by a series of biochemical experiments using PrP chimeras containing the H2H3 domain. We demonstrated that two rabbit PrP chimeras we designed (rabbit chimera and chimera R) did form amyloid fibrils with different structural features in absence and presence of crowding agents. By contrast, the rabbit PrP forms amyloid fibrils with same structural features in absence and presence of crowding agents [27]. We also demonstrated that two human PrP chimeras we designed (human chimera and chimera H) did form amyloid fibrils with same structural features in absence and presence of crowding agents, which is in agreement with those observed in human PrP fibrils [25], [27]. Furthermore, we found that PK resistance of the fibrils from the rabbit chimera is different from that observed in rabbit PrP fibrils [27] but PK resistance of the fibrils from the human chimera is similar to that observed in human PrP fibrils [25], [27]. We then investigated amyloid formation of rabbit PrP-H2H3 and human PrP-H2H3. To our surprise, macromolecular crowding accelerated the nucleation step of fibril formation of both rabbit PrP-H2H3 and human PrP-H2H3, and both rabbit PrP-H2H3 and human PrP-H2H3 formed amyloid fibrils with β-sheet-rich conformation from the native state which has predominant α-helix conformation, which are similar to those of the human PrP [27], [28]. Our data indicate that the structure of amyloid fibrils formed by rabbit PrP-H2H3 is different from that formed by the rabbit PrP [27] while the structure of amyloid fibrils formed by human PrP-H2H3 is similar to that formed by human PrP [25], [27]. All the results above suggest that the amino acids beyond PrP-H2H3 (herein PrP-B1H1B2 domain) have a remarkable effect on fibrillization of the rabbit PrP but almost no effect on the human PrP. In other words, not only the H2H3 domain could play an important role, but also the B1H1B2 domain could take part in fibrillization of the rabbit PrP. Our conclusion that the H2H3 domain plays an important role in PrP fibrillization is in agreement with previously published work [29]–[31], [44], [45]. Rabbit/human chimera formed amyloid fibrils with the same structural features as those of chimera R/H, and the difference between Rabbit/human chimera and chimera R/H is the N-terminal flexibly disordered tail, indicating that an N-terminal flexibly disordered tail of PrP is not important for fibrillization of the rabbit/human PrPs.

First, this paper demonstrated that the B1H1B2 domain causes the difference between fibrillization of the rabbit PrP and the human PrP. Therefore, the present study provides an explanation of how domain replacement affects fibril formation of prion proteins from different species, much better than our previous studies [27], [28]. Second, this paper employed ThT binding assays, Sarkosyl-soluble SDS-PAGE, CD, FTIR, and TEM, similar to our previous studies [28], [33]–[35], but designed four novel PrP chimeras. Therefore, the present study presents a new finding concerning the influence of the amino acids among and beyond PrP-H2H3 on fibrillization of prion proteins from different species, as compared with our previous studies [27], [28].

**Figure 10:**
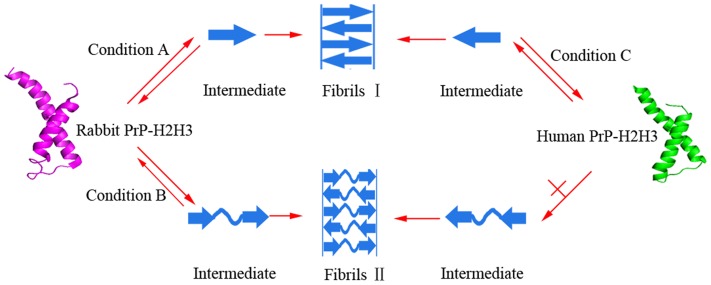
A hypothetical model to explain why amyloid fibrils formed by the rabbit PrP and the human PrP have different structural features. Some chimeras containing rabbit PrP-H2H3 domain could form fibrils I and fibrils II with different structures under different conditions (Conditions A and B), but PrPs containing human PrP-H2H3 domain could only form fibrils I under all conditions (Condition C). A single minimum around 218 nm is observed for fibrils I with β-sheet-rich conformation, but a single minimum around 210 nm is observed for fibrils II with random coils and less β-sheet conformation.

Based on our data and the reported results [27], [28], we propose a valuable hypothetical model to explain why amyloid fibrils formed by the rabbit PrP and the human PrP have different structural features (Fig. 10). Some chimeras containing rabbit PrP-H2H3 domain (rabbit chimera and chimera R) could form fibrils I and fibrils II with different structures under different conditions, but PrPs with human PrP-H2H3 domain (such as wild-type human PrP, human chimera, and chimera H) could only form fibrils I under all conditions and wild-type rabbit PrP could only form fibrils II under all conditions. A single minimum around 218 nm or a single amide I′ band around 1630 cm^−1^ is observed for fibrils I with β-sheet-rich conformation, but a single minimum around 210 nm or two amide I′ bands around 1650 cm^−1^ and 1630 cm^−1^ are observed for fibrils II with random coils and less β-sheet conformation.

In conclusion we have shown that: (i) the presence of a strong crowding agent dramatically promoted fibril formation of four PrP chimeras we designed, and amyloid fibrils from human chimera had biochemical features similar to those from the human PrP; (ii) amyloid fibrils from rabbit chimera had biochemical features in crowded physiological environments different from those from the rabbit PrP. This work is novel as demonstrates that certain protein domains have a significant effect on fibril formation of PrP from rabbit but almost no effect on human PrP, and has the following biochemical and biomedical implications. First, our findings can help to explain why amyloid fibrils formed by the rabbit PrP and the human PrP have different secondary structures and why macromolecular crowding has different effects on fibrillization of PrPs from different species. Second, our findings may help to better understand why human PrP *in vivo* tends to form fibril deposits associated with serious infectious diseases while the rabbit PrP does not and thus is unlikely that will cause prion diseases.

## Supporting Information

Figure S1 **Effects of macromolecular crowding on amyloid formation of chimeras H and R.** Chimera H (A) and chimera R (B) in the absence and presence of Ficoll 70, monitored by ThT fluorescence. All experiments were repeated at least three times. The final concentrations of all prion proteins were 10 µM. The crowding agent concentrations were 0 (open square) and 200 g/l (solid triangle), respectively. A sigmoidal equation was fitted to the data and the solid lines represented the best fit. The insets in B show the kinetics of amyloid formation of chimera R in the presence of Ficoll 70 more clearly.
(DOC)

Table S1 The primers used to replace the human PrP-H2H3 by the rabbit PrP-H2H3.
(DOC)

Table S2 The primers used to replace the rabbit PrP-H2H3 by the human PrP-H2H3.
(DOC)

Table S3 The primers used to construct chimera H in which the human PrP-B1H1B2 (β-strand 1, α-helix 1, and β-strand 2) was replaced by the rabbit PrP-B1H1B2.
(DOC)

Table S4 The primers used to construct chimera R in which the rabbit PrP-B1H1B2 (β-strand 1, α-helix 1, and β-strand 2) was replaced by the human PrP-B1H1B2.
(DOC)
