# Supplementary material for: How Does Domain Replacement Affect Fibril Formation of the Rabbit/Human Prion Proteins
Source: PLoS One. 2014 Nov 17;9(11):e113238. doi: 10.1371/journal.pone.0113238 (PMC4234653; doi:10.1371/journal.pone.0113238)
Supplement: Table S1 — The primers used to replace the human PrP-H2H3 by the rabbit PrP-H2H3. (DOC) [file pone.0113238.s002.doc]

| SN174S | 5’CAGCAACCAGAACAGCTTTGTGCACGACTG3’ |
| --- | --- |
| AN174S | 5’CAGTCGTGCACAAAGCTGTTCTGGTTGCTG3’ |
| SI184V | 5’GTCAATATCACAGTCAAGCAGCAC3’ |
| AI184V | 5’GTGCTGCTTGACTGTGATATTGAC3’ |
| SV203I/M205I | 5’GAGACCGACATTAAGATAATGGAGCGCG3’ |
| AV203I/M205I | 5’CGCGCTCCATTATCTTAATGTCGGTCTC3’ |
| SE219Q/R220Q | 5’CACCCAGTACCAGCAGGAATCTCAG3’ |
| AE219Q/R220Q | 5’CTGAGATTCCTGCTGGTACTGGGTG3’ |
| SY225A | 5’AATCTCAGGCCGCTTACCAGAGAG3’ |
| AY225A | 5’CTCTCTGGTAAGCGGCCTGAGATT3’ |
| SG229A | 5’GCTTACCAGAGAGCATAGAGCTGAGAAT3’ |
| AG229A | 5’ATTCTCAGCTCTATGCTCTCTGGTAAGC3’ |
