# Supplementary material for: How Does Domain Replacement Affect Fibril Formation of the Rabbit/Human Prion Proteins
Source: PLoS One. 2014 Nov 17;9(11):e113238. doi: 10.1371/journal.pone.0113238 (PMC4234653; doi:10.1371/journal.pone.0113238)
Supplement: Table S2 — The primers used to replace the rabbit PrP-H2H3 by the human PrP-H2H3. (DOC) [file pone.0113238.s003.doc]

| SS173N | 5’TACAGCAACCAGAACAACTTCGTGCACGAC3’ |
| --- | --- |
| AS173N | 5’GTCGTGCACGAAGTTGTTCTGGTTGCTGTA3’ |
| SV183I | 5’GTCAACATCACGATCAAGCAGCACACG3’ |
| AV183I | 5’CGTGTGCTGCTTGATCGTGATGTTGAC3’ |
| SI202V/I204M | 5’GAGACCGACGTCAAGATGATGGAGCGCG3’ |
| AI202V/I204M | 5’CGCGCTCCATCATCTTGACGTCGGTCTC3’ |
| SQ218E/Q219R | 5’ATCACGCAGTACGAGAGGGAGTCCCAGGC3’ |
| AQ218E/Q219R | 5’GCCTGGGACTCCCTCTCGTACTGCGTGAT3’ |
| SA224Y | 5’GAGTCCCAGGCCTACTACCAGAGGGCG3’ |
| AA224Y | 5’CGCCCTCTGGTAGTAGGCCTGGGACTC3’ |
| SR229 | 5’ACTACCAGAGGGGGTCATAATTCGAGCTCCGT3’ |
| AR229 | 5’ACGGAGCTCGAATTATGACCCCCTCTGGTAGT3' |
| SR231 | 5’AGAGGGGGTCATCATAGGAGCTCCGTCGAC3’ |
| AR231 | 5’GTCGACGGAGCTCCTATGATGACCCCCTCT3’ |
