# Supplementary material for: How Does Domain Replacement Affect Fibril Formation of the Rabbit/Human Prion Proteins
Source: PLoS One. 2014 Nov 17;9(11):e113238. doi: 10.1371/journal.pone.0113238 (PMC4234653; doi:10.1371/journal.pone.0113238)
Supplement: Table S3 — The primers used to construct chimera H in which the human PrP-B1H1B2 (β-strand 1, α-helix 1, and β-strand 2) was replaced by the rabbit PrP-B1H1B2. (DOC) [file pone.0113238.s004.doc]

| SI138L | 5’ TGCCGAAATGTATGAGGGGCCTGCTCAT 3’ |
| --- | --- |
| AI138L | 5’ ATGAGCAGGCCCCTCATACATTTCGGCA 3’ |
| SS143N | 5’ ACATTTCGGCAATGACTATGAGG 3’ |
| AS143N | 5’ CCTCATAGTCATTGCCGAAATGT 3’ |
| SH155Y | 5’ GTGAAAACATGTACCGTTACCCC 3’ |
| AH155Y | 5’ GGGGTAACGGTACATGTTTTCAC 3’ |
| SM166V/E168Q | 5’ CTACAGGCCCGTGGATCAGTACAGCAAC 3’ |
| AM166V/E168Q | 5’ GTTGCTGTACTGATCCACGGGCCTGTAG 3’ |
