# Supplementary material for: How Does Domain Replacement Affect Fibril Formation of the Rabbit/Human Prion Proteins
Source: PLoS One. 2014 Nov 17;9(11):e113238. doi: 10.1371/journal.pone.0113238 (PMC4234653; doi:10.1371/journal.pone.0113238)
Supplement: Table S4 — The primers used to construct chimera R in which the rabbit PrP-B1H1B2 (β-strand 1, α-helix 1, and β-strand 2) was replaced by the human PrP-B1H1B2. (DOC) [file pone.0113238.s005.doc]

| SL137I | 5’ CCGAAGTGGATGATGGGCCTGCTCATGG 3’ |
| --- | --- |
| AL137I | 5’ CCATGAGCAGGCCCATCATCCACTTCGG 3’ |
| RSN142S | 5’ATCCACTTCGGCAGCGACTACGAGGAC 3’ |
| RAN142S | 5’ GTCCTCGTAGTCGCTGCCGAAGTGGAT 3’ |
| SY154H | 5’GGGAGAACATGCACCGGTACCCC 3’ |
| AY154H | 5’ GGGGTACCGGTGCATGTTCTCCC 3’ |
| RS165-167 | 5’ ACTACAGGCCCATGGACGAGTACAGCAACC 3’ |
| RA165-167 | 5’ GGTTGCTGTACTCGTCCATGGGCCTGTAGT 3’ |
