# Supplementary material for: How Does Domain Replacement Affect Fibril Formation of the Rabbit/Human Prion Proteins
Source: PLoS One. 2014 Nov 17;9(11):e113238. doi: 10.1371/journal.pone.0113238 (PMC4234653; doi:10.1371/journal.pone.0113238)
Supplement: Supplementary file 5 [file pone.0113238.s001.doc]

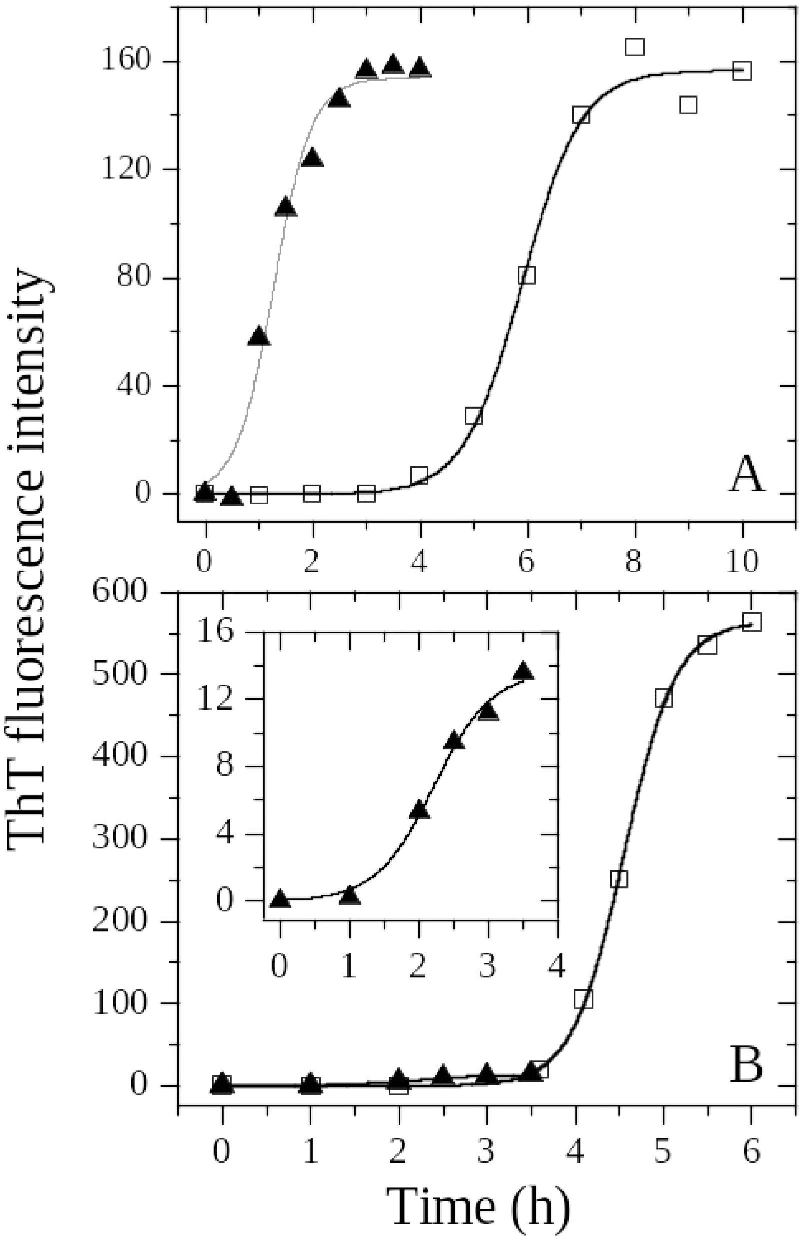
ThT fluorescence intensity
160
120
80
40
0
A
0
2
4
6
8
10
600
500
400
300
200
100
0
16
12
8
4
0
0
1
2
3
4
B
0
1
2
3
4
5
6
Time (h)
